# Desperately seeking…Models to find the right partner and the best use for checkpoint inhibitors

**DOI:** 10.1038/s41416-018-0353-x

**Published:** 2018-12-11

**Authors:** Francesco Bertolini

**Affiliations:** Laboratory of Hematology-Oncology, European Institute of Oncology, Milan, Italy

## Abstract

Immune checkpoint inhibitors have shown unprecedented clinical activity in a variety of cancer types, but only in a fraction of patients. Promising in vivo synergies have been demonstrated between checkpoint inhibitors and other drugs and/or chemotherapy or radiotherapy, and effective models are now needed to select the best combinatorial regimen and disease setting.

## Main

Immune checkpoint inhibitors, such as monoclonal antibodies that target programmed cell death protein 1 (PD-1) or its ligand PD-L1, are currently used for the therapeutic management of a wide spectrum of neoplastic diseases. At the present time, however, checkpoint inhibitors provide long-term and durable remission in only a minority of patients, and side effects are common. For this reason, intense preclinical and clinical research efforts are currently ongoing to improve the efficacy of checkpoint inhibitors, by using them in conjunction with small molecules, antibodies and/or chemotherapy or radiotherapy. The aims of this combinatorial approach include, amongst others, the possibility of generating new immunogenic tumour neoantigens; inhibiting subpopulations of immune suppressor cells in a systemic and/or tumour-specific manner; and mobilising new, activated and more effective immune cells into the tumour microenvironment^1^.

In this issue of the *British Journal of Cancer*, Wu et al.^2^ show that combination of the tyrosine kinase inhibitor (TKI) sunitinib with an anti-PD-L1 checkpoint inhibitor improved overall survival in a renal cancer cell model, compared with the use of either drug alone. These data support the hypothesis that inhibiting vascular endothelial growth factor (VEGF), which is known to promote several suppressive immune cell populations by signalling through a tyrosine kinase, can increase the efficacy of checkpoint inhibitors^3^. The authors also describe that an anti-PD-L1 checkpoint inhibitor synergised with the chemotherapy drug paclitaxel, in a triple negative breast cancer (TNBC) model^2^. Interestingly, these data support the hypothesis from the Semenza laboratory^4^ that chemotherapy might increase the expression of PD-1 in TNBC cells, with the addition of a checkpoint inhibitor improving the efficacy of paclitaxel. Similarly, a recent study has demonstrated synergy between checkpoint inhibitors and several types and dosages of chemotherapy in models of TNBC and lymphoma, possibly owing to the profound effects of the chemotherapy on circulating and tumour-infiltrating, suppressive, antigen-presenting and effector immune cell populations^5^.

### Reducing overprediction

The significance of the Wu et al. paper^2^ is not limited to the demonstration of synergy between checkpoint inhibitors and other drugs. Also notable in their study are the models that were used to simulate and investigate therapeutic strategies in human patients with unresected, local or metastatic disease, as well as in neoadjuvant and adjuvant settings. This comprehensive approach has relevant merit, because preclinical studies that were previously limited to models mimicking an unresected, local subcutaneous disease have generated hype that has subsequently led to multiple failures in clinical trials. These failures could possibly have been prevented by the preclinical use of orthotopic models with features that more closely resembled those of human neoplastic diseases^6^. Looking in more detail, the comprehensive approach reported by Wu et al.^2^ demonstrated that, in the renal cancer model, checkpoint inhibitors plus an anti-VEGFR TKI were effective in the postsurgical adjuvant setting, but ineffective in the unresected primary tumour and in late-stage metastatic disease. In TNBC, a checkpoint inhibitor alone was found to be effective in the adjuvant setting, whereas checkpoint inhibitors synergised with paclitaxel (with or without an anti-VEGF agent) in the neoadjuvant model.

Results from recent clinical trials have shown that combinatorial therapies such as the use of an anti-VEGFR TKI plus checkpoint inhibitors are effective in advanced and/or metastatic renal cancer, albeit with discouraging toxicities observed after the association of pembrolizumab with pazopanib or nivolumab with sunitinib, and more manageable toxicities observed after the administration of pembrolizumab together with axitinib (see ref. ^7^ for a review). In view of the present data from Wu et al.^2^, however, clinical trials in the postsurgical, adjuvant setting are also encouraged^7^.

Combinatorial therapies including chemotherapy plus checkpoint inhibitors have shown benefit over a single therapy approach in recent randomised clinical trials in metastatic non–small cell lung cancer (NSCLC; an overall survival advantage has been reported in ref. ^8^) and advanced TNBC (a progression-free survival advantage was reported in ref. ^9^). Interestingly, in another randomised trial that enroled patients with advanced NSCLC, the addition of a checkpoint inhibitor to an anti-VEGF monoclonal antibody plus chemotherapy significantly improved progression-free survival and overall survival^10^.

### Outlook

Taken together, these preclinical and clinical data suggest that checkpoint inhibitors might successfully be included in combinatorial regimens aimed at improving the percentage of patients who receive a clinical benefit and prolonging the duration of this benefit for several different types of cancer. What is still missing, however, in spite of intense research in the field, is a clinically effective biomarker that is able to identify patients who are likely to respond to these new combinatorial regimens and/or to enable severe toxicities to be avoided. Again, lessons from the past suggest that 'overprediction' of positive results from preclinical studies can be avoided only by using models that are able to fully recapitulate the human neoplastic disease with its orthotopic characteristics—including the tumour microenvironment and a functional immune system—before or after surgical resection.

## References

[1] Galluzzi L, Chan TA, Kroemer G, Wolchok JD, López-Soto A (2018). The hallmarks of successful anticancer immunotherapy. Sci. Transl. Med..

[2] Wu FTH, Xu P, Chow A, Man S, Kruger J, Khan KA (2018). Pre-and post-operative anti-PD-L1 plus anti-angiogenic therapies in mouse breast or renal cancer models of micro- or macro-metastatic disease. Br. J. Cancer.

[3] Khan KA, Kerbel RS (2018). Improving immunotherapy outcomes with anti-angiogenic treatments and vice versa. Nat. Rev. Clin. Oncol..

[4] Samanta D, Park Y, Ni X, Li H, Zahnow CA, Gabrielson E (2018). Chemotherapy induces enrichment of CD47^+^/CD73^+^/PDL1^+^ immune evasive triple-negative breast cancer cells. Proc. Natl. Acad. Sci. USA.

[5] Orecchioni S, Talarico G, Labanca V, Calleri A, Mancuso P, Bertolini F (2018). Vinorelbine, cyclophosphamide and 5-FU effects on the circulating and intratumoural landscape of immune cells improve anti-PD-L1 efficacy in preclinical models of breast cancer and lymphoma. Br. J. Cancer.

[6] Kerbel RS (2015). A decade of experience in developing preclinical models of advanced- or early-stage spontaneous metastasis to study antiangiogenic drugs, metronomic chemotherapy, and the tumor microenvironment. Cancer J..

[7] Lee CH, Motzer R (2018). Combination VEGFR/immune checkpoint inhibitor therapy: a promising new treatment for renal cell carcinoma. Br. J. Cancer.

[8] Gandhi L, Rodríguez-Abreu D, Gadgeel S, Esteban E, Felip E, De Angelis F (2018). KEYNOTE-189 Investigators. Pembrolizumab plus chemotherapy in metastatic non-small-cell lung cancer. N. Engl. J. Med..

[9] Schmid P, Adams S, Rugo HS, Schneeweiss A, Barrios CH, Iwata H (2018). IMpassion130 Trial Investigators. Atezolizumab and Nab-Paclitaxel in advanced triple-negative breast cancer. N. Engl. J. Med..

[10] Socinski MA, Jotte RM, Cappuzzo F, Orlandi F, Stroyakovskiy D, Nogami N (2018). IMpower150 Study Group. Atezolizumab for first-line treatment of metastatic nonsquamous NSCLC. N. Engl. J. Med..

