# Correction: Desperately seeking…Models to find the right partner and the best use for checkpoint inhibitors

**DOI:** 10.1038/s41416-019-0554-y

**Published:** 2019-08-19

**Authors:** Francesco Bertolini

**Affiliations:** Laboratory of Hematology-Oncology, European Institute of Oncology, Milan, Italy

**Correction to**: *British Journal of Cancer* (2019) **120**, 139–140; 10.1038/s41416-018-0353-x; published online 11 December 2018.

Since the publication of this paper, the author noticed that the funding information was not complete. The correct funding information is now shown in the Funding section.

**Funding**

FB is funded by the AIRC—Fondazione AIRC per la Ricerca sul Cancro | AIRC.

