# Correction: Desperately seeking…Models to find the right partner and the best use for checkpoint inhibitors

**DOI:** 10.1038/s41416-019-0591-6

**Published:** 2019-09-24

**Authors:** Francesco Bertolini

**Affiliations:** Laboratory of Hematology-Oncology, IEO European Institute of Oncology IRCCS, Milan, Italy

**Correction to**: *British Journal of Cancer* (2019) **120**, 139–140; 10.1038/s41416-018-0353-x; published online 11 December 2018.

Since the publication of this paper, the author noticed that the affiliation information was not complete. The correct affiliation is now shown below.

Laboratory of Hematology-Oncology, IEO European Institute of Oncology IRCCS, Milan, Italy

